# Nomogram Based on CT Radiomics Features Combined With Clinical Factors to Predict Ki-67 Expression in Hepatocellular Carcinoma

**DOI:** 10.3389/fonc.2022.943942

**Published:** 2022-07-06

**Authors:** Cuiyun Wu, Junfa Chen, Yuqian Fan, Ming Zhao, Xiaodong He, Yuguo Wei, Weidong Ge, Yang Liu

**Affiliations:** ^1^ Cancer Center, Department of Radiology, Zhejiang Provincial People’s Hospital (Affiliated People’s Hospital, Hangzhou Medical College), Hangzhou, China; ^2^ Department of Clinical Pathology, Graduate School, Hebei Medical University, Shijiazhuang, China; ^3^ Cancer Center, Department of Pathology, Zhejiang Provincial People’s Hospital (Affiliated People’s Hospital, Hangzhou Medical College), Hangzhou, China; ^4^ Precision Health Institution, General Electrical Healthcare, Hangzhou, China; ^5^ Cancer Center, Department of Ultrasound Medicine, Zhejiang Provincial People’s Hospital (Affiliated People’s Hospital, Hangzhou Medical College), Hangzhou, China

**Keywords:** hepatocellular carcinoma, Ki-67, radiomics, computed tomography, models, nomograms

## Abstract

**Objectives:**

The study developed and validated a radiomics nomogram based on a combination of computed tomography (CT) radiomics signature and clinical factors and explored the ability of radiomics for individualized prediction of Ki-67 expression in hepatocellular carcinoma (HCC).

**Methods:**

First-order, second-order, and high-order radiomics features were extracted from preoperative enhanced CT images of 172 HCC patients, and the radiomics features with predictive value for high Ki-67 expression were extracted to construct the radiomic signature prediction model. Based on the training group, the radiomics nomogram was constructed based on a combination of radiomic signature and clinical factors that showed an independent association with Ki-67 expression. The area under the receiver operating characteristic curve (AUC), calibration curve, and decision curve analysis (DCA) were used to verify the performance of the nomogram.

**Results:**

Sixteen higher-order radiomic features that were associated with Ki-67 expression were used to construct the radiomics signature (AUC: training group, 0.854; validation group, 0.744). In multivariate logistic regression, alfa-fetoprotein (AFP) and Edmondson grades were identified as independent predictors of Ki-67 expression. Thus, the radiomics signature was combined with AFP and Edmondson grades to construct the radiomics nomogram (AUC: training group, 0.884; validation group, 0.819). The calibration curve and DCA showed good clinical application of the nomogram.

**Conclusion:**

The radiomics nomogram developed in this study based on the high-order features of CT images can accurately predict high Ki-67 expression and provide individualized guidance for the treatment and clinical monitoring of HCC patients.

## Introduction

Hepatocellular carcinoma (HCC) is one of the most common malignancies that harms human life. Globally, it is the third leading cause of cancer-related deaths in men (1), causing a considerable socioeconomic burden (2). Although hepatectomy is the primary treatment for HCC, the 5-year survival rate is only 14–18% (3) due to a high incidence of postoperative recurrence (4).

Ki-67 is a protein present in the nucleus that is expressed during cell proliferation (5). Ki-67 expression is believed to be associated with the therapeutic effect and prognosis of malignant tumors (6, 7). The Ki-67 proliferation index (PI) has been widely used as a prognostic indicator of many malignancies, such as glioma, breast cancer, lung cancer, and liver cancer (8–11). Ki-67 proliferation is closely related to tumor growth rate, and high Ki-67 expression has been shown to be associated with poor overall survival and relapse-free survival rate of HCC patients (7, 12). Owing to its prognostic significance, the detection of the expression status of Ki-67 is important for treating HCC patients. Currently, immunohistochemical methods are used to assess Ki-67 expression status in tumor specimens obtained from surgery or biopsy. However, biopsy samples are unrepresentative of the entire tumor. Additionally, Ki-67 expression is not routinely assessed in many centers (13). Therefore, the development of markers that can predict the status of Ki-67 is a key imperative to guide individualized treatment decision-making and for postoperative monitoring of patients with HCC.

Radiomics is a recently developed technology that can quantify tumor characteristics based on a large amount of high-throughput data. Radiomics can help predict the tumor phenotype and heterogeneity (14, 15) and provide information on the biological behavior and pathophysiology of tumors (14). The radiomics approach has been used for the diagnosis, treatment, and prognostic assessment in the context of various tumors (16–19). At present, only a few studies have assessed the application of radiomics for predicting Ki-67 expression in HCC patients. Wu et al. (11) used computed tomography (CT) images to delineate two-dimensional (2D) regions of interest with the maximum diameter of lesions and extracted texture features to predict high Ki-67 expression. Other studies have employed magnetic resonance imaging (MRI) to analyze the correlation of texture features and histograms with Ki-67 status (20, 21). However, these studies only extracted first-order features (histogram) or (and) second-order texture features to construct models. Additionally, these studies had a relatively small sample size. Fan et al. (22) used MRI analysis radiomic features to predict the expression status of Ki-67, but they did not quantify the risk probability of Ki-67 expression. Some studies have proposed that use of the Laplacian of Gaussian (LOG) filter to transform features for CT texture analysis can reduce photon noise and enhance edge detection (23). Feature extraction using the wavelet transform is an important part of the segmentation method, which may help unravel the tumor features unobservable in the original image (24, 25). Most of these features qualify the definition of features described by the Imaging Biomarker Standardization Initiative (IBSI) (25). According to existing studies, radiomic features [first-order features or (and) second-order features] can be used as a potential prognostic marker for Ki-67 expression in HCC patients (11, 20, 21). However, it has not been found that the expression of Ki-67 in HCC patients can be predicted based on the radiomic features (including first-order, second-order, and high-order features) described by IBSI and extracted from contrast-enhanced CT images.

In this study, we used contrast-enhanced CT images to outline the three-dimensional (3D) volume of tumors, extract high-order features (such as LOG, wavelet filters processing) as well as first- and second-order features. Then, we established and verified a nomogram based on a combination of quantitative radiomics features and clinical factors and explored the correlation between radiomics and Ki-67 expression in HCC. The ability of the nomogram to predict the probability of Ki-67 expression in individual patients and its use for risk stratification was assessed.

## Methods and Materials

### Patients

Our institutional ethics committee approved this retrospective study, and the requirement for written informed consent was waived off. The data of 329 patients with HCC confirmed by histopathological examination of surgical specimens between June 2015 and January 2022 were identified for this study. The inclusion criteria were: (1) age ≥18 years; (2) pathologically confirmed HCC with definite Ki-67 proliferation index and Edmondson grade; (3) enhanced CT examination performed within 1 month before surgery; (4) no previous history of tumor treatment; (5) in patients with multiple lesions, the largest lesion consistent with the pathological and immunohistochemical diagnosis was selected. The exclusion criteria were: (1) poor image quality (n = 19); (2) incomplete CT images, clinical, or pathological data (n = 75); and (3) history of anti-tumor therapy before operation (n = 63). After the exclusion of 157 ineligible patients, 172 subjects were retrospectively enrolled in the study (**Supplementary Figure S1**). Ordered by CT examination time (26), patients were divided into two groups in a 7:3 ratio, i.e., a training group for model construction (n = 120) and a validation group for validation of model performance (n = 52).

### Data Collection and Acquisition of CT Images

Demographic characteristics and clinicopathological information were collected for each patient, namely, age, sex, serum hepatitis B surface antigen (HBsAg), alpha-fetoprotein (AFP) level, and Edmondson grade. Serum AFP level was included as a categorical variable based on the threshold value (20 μg/L).

**Supplementary Table S1** illustrates the parameters of enhanced CT image scanning, and **Supplementary Material I** describes the specific image acquisition information. Two radiologists (radiologist A with 10 years and radiologist B with 19 years of experience in diagnosing liver tumors) independently evaluated the preoperative CT images of each patient, namely, tumor size, cirrhosis, tumor capsule, and tumor margin. In the event of any disagreement, the CT image features were reevaluated and a consensus was reached. The radiologists were blinded to the clinicopathological information.

### Histological and Immunohistochemistry

All specimens were fixed in 3.7% neutral formaldehyde solution, dehydrated conventionally, paraffin-embedded, and cut into 4-μm thick sections. Immunohistochemistry (IHC) was used to detect Ki-67 proliferation status using Ventana Benchmark Ultra automated immunohistochemical staining (Roche Ventana, Inc.). B Tumor cells with brown nuclei were thought to have positive Ki-67 expression. The positive percentage of tumor nuclei was counted as PI. According to Ki-67 PI, HCC lesions were divided into two groups: the high expression group (PI ≥20%) and the low expression group (PI <20%).

### Tumor Segmentation and Feature Extraction

Each CT image (including arterial phase and portal vein phase) was preprocessed before tumor segmentation, including: (1) Voxel size resampling: a linear interpolation method was used to resample CT images to 1 × 1 × 1 mm^3^ voxel size to achieve image standardization. (2) Gray-level normalization and discretization to order 32 to improve the robustness of features. The 3D volume of interest (VOI) of the lesion was manually delineated using ITK-SNAP software (http://www.itksnap.org/). The CT images were independently evaluated by radiologists A and B, with radiologist A drawing the tumor boundaries and radiologist B validating them. The two radiologists were unaware of the study.

Features were extracted from original and derived images using the open-source package PyRadiomics (https://github.com/Radiomics/pyradiomics), and derived images using wavelet and LOG filters (**Supplementary Material II**). Additionally, shape features were extracted from the original images. The extracted radiomic features included morphological features, first-order statistical features, and texture features. All image types and radiomic feature types are shown in **Supplementary Table S2**. As detailed in **Supplementary Material II**, Z-score normalization was used to standardize the data before feature extraction.

The repeatability of feature extraction was assessed using inter- and intra-class correlation coefficients (ICCs). Radiologists A and B randomly selected 30 lesions from the two phases to delineate VOI, respectively. The features extracted by the two radiologists were compared to calculate the inter-observer reproducibility. Then, radiologist A segmented the 30 VOIs again 7 days later and extracted features, and the features extracted were compared with the first time to calculate the intra-observer reproducibility. An ICC value greater than 0.8 was considered indicative of good consistency of feature extraction (27).

### Construction of the Radiomics Signature

To select the most optimal radiomic features, analysis of variance (ANOVA), Mann–Whitney U-Test, correlation analysis, and gradient boosting decision tree (GBDT) were used for dimension reduction in the training set. The detailed steps are shown in **Supplementary Material III**. The final radiomic features selected from the training group were multiplied by their respective coefficients to calculate the radiomic score (rad-score) of each patient. The rad-score of the arterial stage (AP), portal vein stage (PVP), and arterial combined portal vein stage (AVP) were calculated, respectively. Additionally, we used first-and second-order (FSO) radiomic features, high-order (HO) radiomic features, and all radiomic features to establish prediction models. The receiver operating characteristic (ROC) curve analysis was performed to evaluate the predictive performance. The Delong test was used to compare the differences between them. The radiomic signature was finally established by selecting radiomic features with the highest area under the ROC curve (AUC).

### Radiomics Nomogram Construction and Evaluation

**Figure 1:**
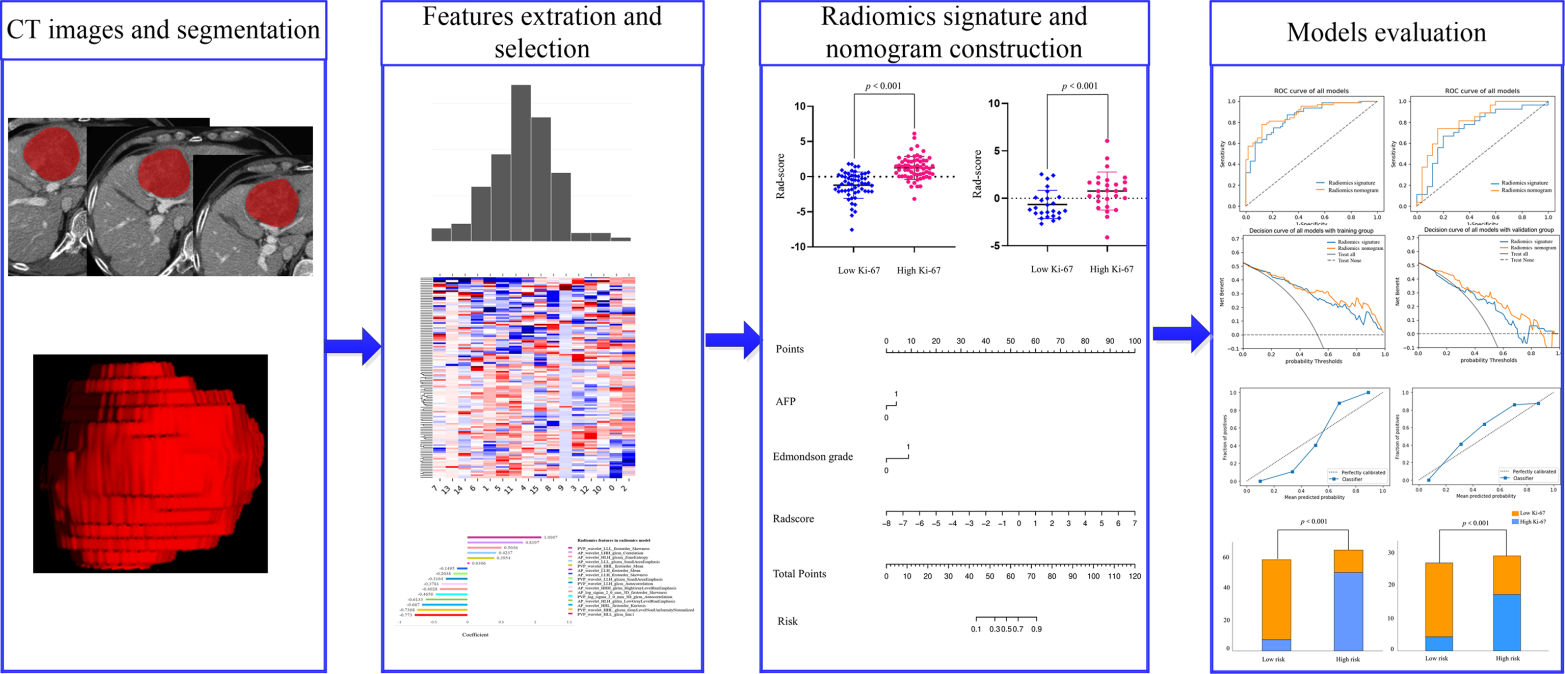
Flowchart for the construction and evaluation of radiomics nomogram.

Variables in the training set were analyzed by univariate logistic regression, and variables associated with a *P*-value of <0.05 were included in multivariate logistic regression analysis to determine independent predictors of high Ki-67 expression. Then, independent predictors along with radiomic signatures were used to establish a radiomics nomogram. AUC was used to evaluate the performance of different models, and the DeLong test was used to compare the differences between them. The calibration curve was used to graphically show the performance of the nomogram (evaluating the consistency between the predicted and actual Ki-67 probabilities). The clinical utility of the nomogram was evaluated using decision curve analysis (DCA) (28). The goodness of fit of the nomogram was analyzed using the Hosmer–Lemeshow (H-L) test (29). *P >*0.05 was considered a good outcome. The risk probability for each patient was calculated according to the nomogram, and the Ki-67 probability was divided into the high-risk group (risk >cut-off value) and the low-risk group (risk <cut-off value), and their pathological Ki-67 results were compared. **Figure 1** shows a flowchart for the construction and evaluation of this nomogram.

### Statistical Analysis

For statistical analysis, SPSS software (version 25.0), MedCalc software (version 19.6), and R software (version 3.4) were used. An independent-sample *t*-test or Mann–Whitney U test was used for the analysis of continuous variables. The chi-square test or Fisher’s exact test was used for categorical variables. The goodness of fit was assessed using the H-L test. The Delong test was used to compare the differences in AUCs. The “rms” software package was used to draw the calibration curves and nomogram, and the DCA was constructed using the “dca. R”. *P*-values of <0.05 were considered indicative of statistical significance.

## Results

### Characteristics of the Study Population

A total of 172 patients were enrolled in this study, and were divided into training (n = 120) and validation (n = 52) groups. There were no significant differences between the training and validation groups with respect to clinical or radiological features (*P >*0.05 for all) (**Supplementary Table S3**). There were significant differences between the high Ki-67 expression and the low Ki-67 expression groups with respect to Edmondson grade, tumor capsule, and tumor margin (*P <*0.05 for all). AFP expression in the high expression group was significantly higher than that in the low expression group (*P <*0.05) (**Table 1**).

**Table 1:** Characteristics of HCC patients in the high and low Ki-67 expression groups.

Variables	Training group (n = 120)			Validation group (n = 52)		
	High expression (n = 63)	Low expression (n = 57)	*P-value*	High expression (n = 27)	Low expression (n = 25)	*P-value*
Age (years)	57.4 ± 11.61	58.84 ± 10.59	0.479	60.93 ± 11.69	61.32 ± 11.26	0.902
Sex			0.855			0.193
Female	6 (9.5%)	6 (10.5%)		5 (18.5%)	1 (4%)	
Male	57 (90.5%)	51 (89.5%)		22 (81.5%)	24 (96%)	
HBs-Ag			0.613			0.853
Negative	12 (19%)	13 (22.8%)		8 (29.6%)	8 (32%)	
Positive	51 (81%)	44 (77.2%)		19 (70.4%)	17 (68%)	
AFP (µg/L)			0.001*			0.053
≤20	19 (30.2%)	34 (59.6%)		7 (25.9%)	13 (52%)	
>20	44 (69.8%)	23 (40.4%)		20 (74.1%)	12 (48%)	
Edmondson grade			0.002*			0.002*
I–II	38 (60.3%)	49 (86%)		13 (48.1%)	22 (88%)	
III–IV	25 (39.7%)	8 (14%)		14 (51.9%)	3 (12%)	
Tumor size			0.665			0.054
≤5 cm	40 (63.5%)	34 (59.6%)		9 (33.3%)	15 (60%)	
>5 cm	23 (36.5%)	23 (40.4%)		18 (66.7%)	10 (40%)	
Cirrhosis			0.13			0.392
Absent	37 (58.7%)	41 (71.9%)		13 (48.1%)	15 (60%)	
present	26 (41.3%)	16 (28.1%)		14 (51.9%)	10 (40%)	
Tumor capsule			0.017*			0.02*
Complete	34 (54%)	42 (75%)		12 (44.4%)	19 (76%)	
Incomplete	29 (46%)	14 (25%)		15 (55.6%)	6 (24%)	
Tumor margin			0.017*			0.001*
Smooth	29 (46%)	38 (67.9%)		7 (25.9%)	18 (72%)	
Non-smooth	34 (54%)	18 (32.1%)		20 (74.1%)	7 (28%)	

HBsAg, serum hepatitis B surface antigen; AFP, alpha-fetoprotein. *p < 0.05.

### Construction of the Radiomics Signature

Extracted from AP, PVP, and AVP images, they included 1,037, 1,037, and 2,074 radiomic features, respectively. The Spearman rank correlation test was used to remove radiomic features with correlation coefficients lower than 0.70. After dimension reduction by GBDT, 9, 11, and 16 features were obtained, respectively. **Supplementary Material III** and **Supplementary Table S4** show the processes and details of the selected features, and **Supplementary Material IV** shows the calculation formula for rad-score.

**Figure 2:**
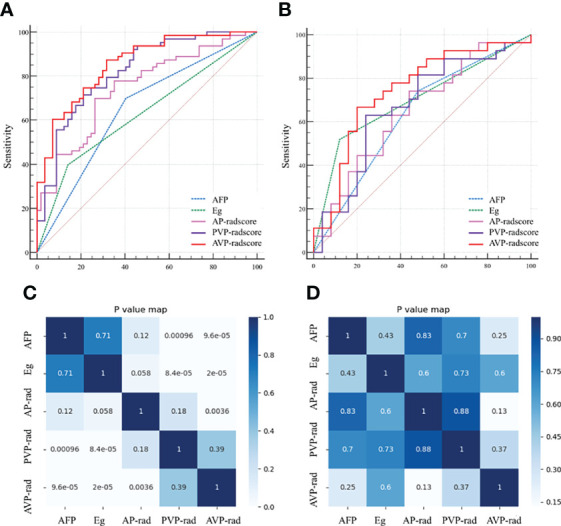
Receiver operating characteristics (ROC) curves of AP, PVP, AVP and clinical independent predictors in the training **(A)** and validation **(B)** groups; Heat maps of these major parameters in the training **(C)** and validation **(D)** groups. AP, arterial phase; PVP, portal venous phase; AVP, arterial phase combined with portal venous phase. AFP, alpha-fetoprotein; Eg, Edmondson grade.

**Figure 3:**
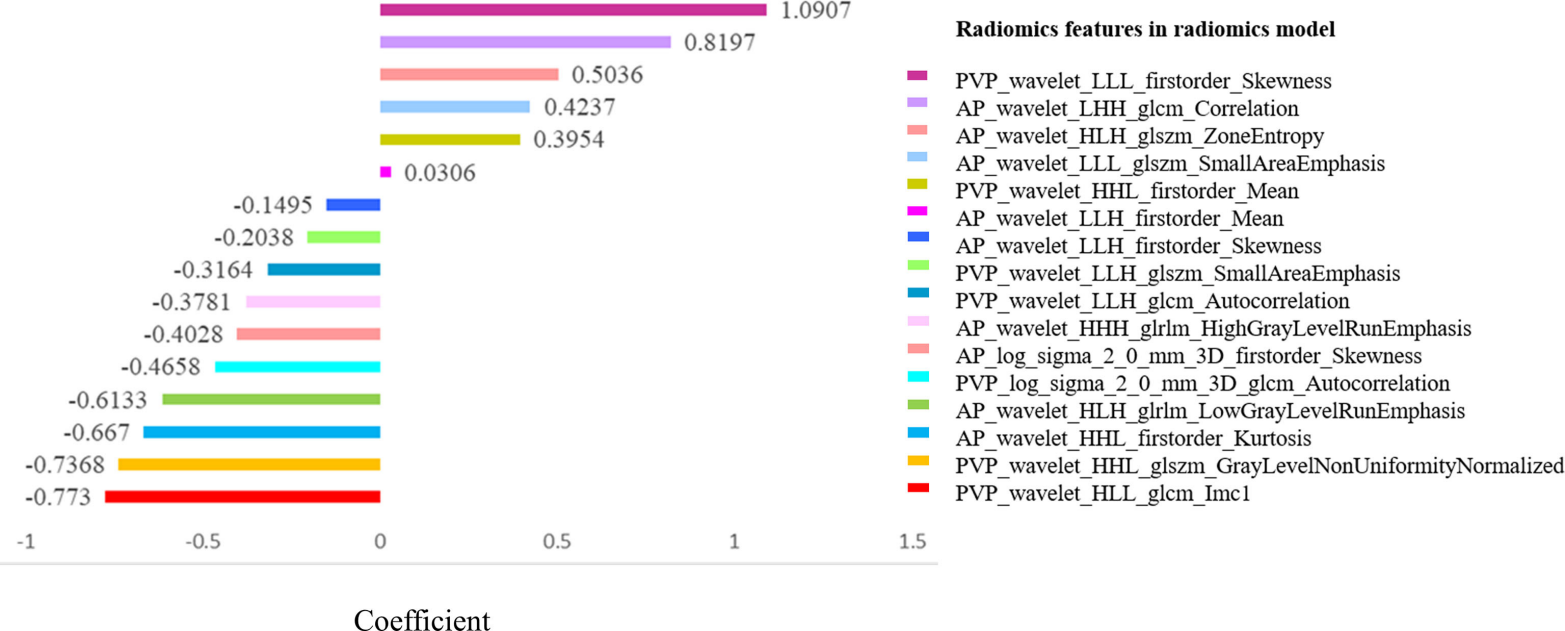
Correlation coefficients of selected radiomic features.

**Figure 4:**
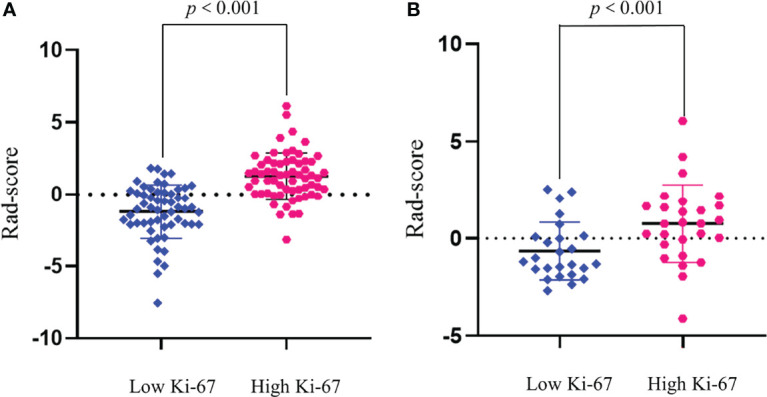
Rad-score between high and low Ki-67 expression groups in the training **(A)** and validation **(B)** groups. Rad-score of the high Ki-67 expression group (red) was significantly higher than that of the low Ki-67 expression group (blue).

We compared the AUC values of rad-score from AP, PVP, and AVP. The AUC values of the rad-score of AVP were the highest (**Supplementary Table S5** and **Figures 2A, B**) [0.854 (0.778–0.912) (training group) and 0.744 (0.604–0.855) (validation group), respectively. The Delong test showed that the AUC value of the AVP rad-score was significantly different from that of the AP rad-score in the training group, but there was no significant difference in other groups (*P >*0.05) (**Figures 2C, D**). Additionally, the AUCs of all radiomic feature models were the highest, which were higher than those of the FSO and HO models (**Supplementary Table S6**). We selected the remaining radiomic features of AVP images, including all radiomic features after dimension reduction to construct a radiomic signature, including 6 first-order features, 4 Gray-Level Cooccurence Matrix (GLCM) features, 2 Gray-Level Run Length Matrix (GLRLM) features, and 4 Gray-Level Size Zone Matrix (GLSZM) features; there were 9 features in AP and 7 features in PVP. **Figure 3** shows the correlation coefficients of these features. The rad-score of the high-expression Ki-67 group was significantly greater than that of the low-expression Ki-67 group (*P <*0.001) (**Figures 4A, B**).

### Development of a Radiomics Nomogram Based on Combination of Radiomics Signature and Clinical Independent Predictors

**Figure 5:**
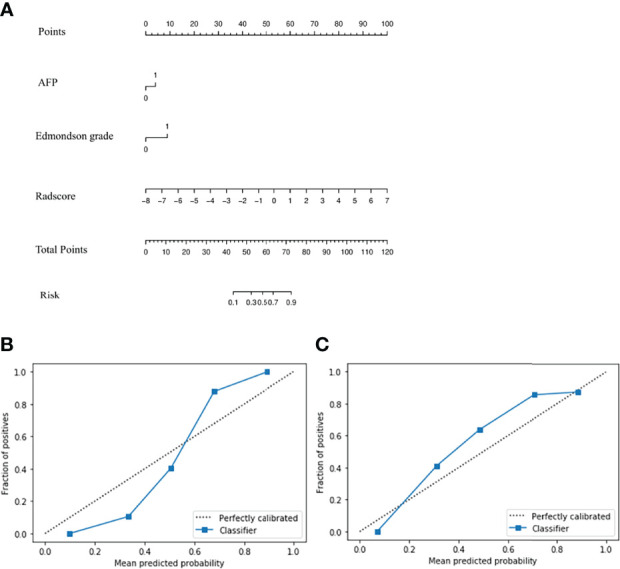
Radiomics nomogram **(A)** Calibration curve of the radiomics nomogram in training **(B)** and validation groups **(C)**.

In univariate regression analysis in the training group, AFP level, tumor capsule, tumor margin, and Edmondson grade showed significant differences between the high and low Ki-67 groups (*P <*0.05) (**Table 2**). On multivariate regression analysis, AFP and Edmondson grade were identified as independent predictors of high-expression of Ki-67 (**Table 2**). **Table 3** and **Figure 2** show the details of these important variables. Hence, the radiomics nomogram was developed using a combination of the radiomic signature, AFP, and Edmondson grade (**Figure 5A**).

**Table 2:** Univariate and multivariate logistic regression analysis of the preoperative clinical and radiological features of training group.

Variables	Univariate logistic regression		Multivariate logistic regression	
	OR (95% CI)	*p*-value	OR (95% CI)	*p*-value
Age	0.988 (0.956–1.021)	0.476		
Sex	1.118 (0.339–3.685)	0.855		
HBs-Ag	1.256 (0.520–3.034)	0.613		
AFP	3.423 (1.610–7.281)	0.001*	2.862 (1.299–6.306)	0.009*
Tumor size	0.850 (0.407–1.776)	0.666		
cirrhosis	1.801 (0.838–3.870)	0.132		
Tumor capsule	2.559 (1.171–5.592)	0.019*	0.840 (0.209–3.375)	0.806
Tumor margin	2.475 (1.171–5.231)	0.018*	1.708 (0.662–4.406)	0.268
Edmondson grade	4.030 (1.635–9.930)	0.002*	2.982 (1.164–7.638)	0.023*

HBsAg, serum hepatitis B surface antigen; AFP, alpha-fetoprotein. *p < 0.05.

**Table 3:** Diagnostic performance of the various models.

Models	Training group (n = 120)			Validation group (n = 52)		
	AUC (95% CI)	Sensitivity	Specificity	AUC (95% CI)	Sensitivity	Specificity
AFP	0.647 (0.555–0.732)	0.698	0.597	0.63 (0.485–0.760)	0.741	0.52
Eg	0.628 (0.535–0.715)	0.397	0.86	0.699 (0.556–0.819	0.519	0.88
Rad-score	0.854 (0.778–0.912)	0.873	0.684	0.744 (0.604–0.855)	0.667	0.8
Nomogram	0.884 (0.813–0.936)	0.778	0.877	0.819 (0.688–0.912)	0.741	0.84

AFP, alpha-fetoprotein; Eg, Edmondson grade.

### Performance Evaluation of the Radiomics Nomogram

The *P-*values of the H-L goodness of fit test were all greater than 0.05 (training group: *P* = 0.643; validation group: *P* = 0.962), indicating a good agreement between the evaluated grade by the calibration curve of the nomogram and the actual grade (**Figures 5B, C**). The nomogram performed well in the Ki-67 grading evaluation.

**Figure 6:**
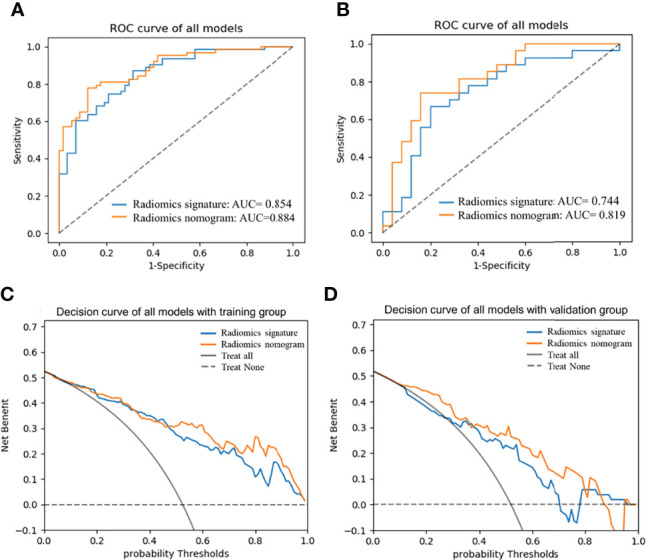
AUCs of radiomics nomogram and radiomic signature in training **(A)** and validation **(B)** groups; Decision curve analysis curve of the nomogram in training **(C)** and validation groups **(D)**. AUC, area under the receiver operating characteristic curve.

The AUC of the radiomics nomogram for the training and validation groups was 0.884 (0.813–0.936) and 0.819 (0.688–0.912) (**Figures 6A, B**) (sensitivity: 0.778 and 0.741, respectively; specificity: 0.877 and 0.84, respectively) (**Table 3**). The AUC value of the radiomics nomogram was greater than that of the clinical model and radiomic signature, and the Delong test showed that the Ki-67 grading performance of the nomogram was better than that of the clinical model (*P <*0.05). There was no significant difference in performance from radiomic signature (*P >*0.05) (**Supplementary Figure S2**), demonstrating the excellent ability of radiomic features in the evaluation of Ki-67 grading.

The DCA was used to assess the utility of the radiomics nomogram based on the area under the decision curve (**Figures 6C, D**). The area under the nomogram (orange) was larger than that of the radiomics signature (blue), and was superior to the “treat all” (solid gray line) or “treat none” (dotted gray line) strategies (**Supplementary Material III**). This demonstrated the good utility of the nomogram of the training and the validation groups for clinical decision-making.

**Figure 7:**
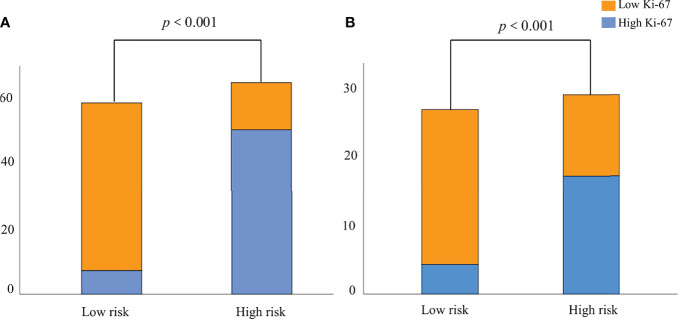
The probability of high Ki-67 expression in the high-risk group was significantly higher than that in the low-risk group in the training **(A)** and validation **(B)** groups.

According to the nomogram (**Figure 5A**), the Ki-67 expression was divided into a high-risk group (risk >0.59288) and a low-risk group (risk <0.59288) based on the optimal cutoff value. As shown in **Figures 7A, B**, there was a significant difference in the number of patients in the risk groups of Ki-67 predicted by the nomogram (*P <*0.001), indicating good discriminative capacity of the nomogram.

## Discussion

In this study, we developed and verified a radiomics nomogram based on CT radiomic features combined with clinical parameters to predict the probability of Ki-67 expression in individual HCC patients. The results demonstrated an excellent performance of the nomogram in predicting Ki-67 expression.

Studies have shown that standardized preprocessing of CT images before feature extraction can significantly reduce the differences between different scanners and different imaging parameters (30). In this study, we conducted standardized preprocessing (including voxel size resampling and gray-level discretization) before the feature extraction. Features extracted using PyRadiomics conform to the feature definition described by the IBSI (25), and were shown to be associated with better reproducibility of CT features and reduced image specification differences (30). In our study, AVP had the highest AUC value. Some studies have showed that the performance of the combined model of multiple sequences is higher than that of a single sequence, indicating that multiple sequences provide more information (26, 31–33). Hence, radiomic features of images, including arterial and portal phases, may provide more important information.

First-order statistics, often called histogram features, are used to describe the voxel intensity distribution of images. Second-order statistics, widely referred to as “texture analysis,” describe the spatial relationships between voxels with similar gray-levels within the lesion (34, 35) that can reflect tumor heterogeneity and complexity (23, 36). These allow for better quantification and assessment of the heterogeneity of tumor texture than first-order features (23). Higher-order statistics entail the application of filters to images, such as wavelets and LOG (34). Feature transformation using a LOG filter can reduce noise and enhance edge detection, and wavelet-transformed features play a crucial role in predicting pathological responses (37). Higher-order features may help reveal tumor features unobservable in the original image (25). In this study, the radiomic model was developed using 16 most discriminative features extracted from higher-order features (including fourteen wavelet transformed features and two LOG transformed features), including 6 first-order features, 4 GLCM features, 2 GLRLM features, and 4 GLSZM features.

In our study, the rad-score of the Ki-67 high-expression group consisting of these 16 features was significantly higher than that of the Ki-67 low-expression group, indicating that the higher the rad-score value, the greater the pixel difference between the image and the greater the tumor heterogeneity (38). High expression of Ki-67 reflects tumor heterogeneity. Wu et al. (11) analyzed CT texture features of tumors at the two-dimensional level to predict the proliferation state of Ki-67. Ye et al. (20) analyzed the texture features of enhanced MRI to predict the expression state of Ki-67, and Hu et al. (21) predicted Ki-67 state with MRI first-order histogram features.

They analyzed the features of images from the first- or second-order features and lacked verification. We used tumor 3D volume to extract features, which provides better morphological information than 2D and better reflects the tumor heterogeneity (34, 39). We developed and verified a radiomics nomogram using higher-order features to explore the correlation between Ki-67 expression and a combination of radiomics and clinical parameters and quantified the risk of high Ki-67 expression.

We extracted the most valuable 16 high-order radiomics features to construct the radiomics model, which showed good performance in predicting Ki-67 expression (AUC: training group, 0.854; validation group, 0.744). Our findings indicate that radiomics can reflect the Ki-67 expression in HCC in a non-invasive manner. In our study, higher AFP levels, incomplete tumor capsule, non-smooth tumor margins, and higher Edmondson grade were more common in the high Ki-67 expression group, which is consistent with previous studies (20, 21, 40, 41). AFP and Edmondson grade were independent predictors of high Ki-67 expression in this study. Higher AFP expression and lower tumor differentiation were shown to be associated with higher HCC invasiveness (42, 43).

Based on a combination of the radiomic signature and independent clinical factors, we constructed the radiomics nomogram to predict the expression status of Ki-67 in individual patients. The nomogram showed the highest predictive performance (AUC: training, 0.884; validation, 0.819) and good calibration capability (*P >*0.05 in the H-L test). Results of the Delong test showed that the nomogram had better predictive performance than clinical factors (*P <*0.05); however, the performance was not significantly different from that of the radiomic signature (*P >*0.05), indicating a significant role of radiomics in predicting the expression of Ki-67. The DCA demonstrated the better clinical value of the radiomics nomogram compared with the radiomic signature. This indicates that clinical factors play a certain complementary role, and the combination of clinical factors has better clinical practicality.

Some limitations of our study should be considered. First, this retrospective study had a potential selection bias with respect to the inclusion of patients with hepatitis B virus-associated HCC. A prospective study including patients with different etiologies must validate these findings. Second, this was a single-center study, and the performance of the nomogram requires external validation. Third, there is currently no unified standard for the expression level of Ki-67 in HCC, and there is no clear consensus on the use of 20% as the cut-off value.

In summary, we developed and validated a radiomics nomogram based on radiomic features and clinical factors, which can quantify the probability of Ki-67 expression, guide individualized treatment and clinical monitoring and show good diagnostic performance and clinical utility.

## Data Availability Statement

The original contributions presented in the study are included in the article/**Supplementary Material**. Further inquiries can be directed to the corresponding authors.

## Ethics Statement

The studies involving human participants were reviewed and approved by the Medical Ethics Committee of Zhejiang Provincial People’s Hospital, Affiliated People’s Hospital, Hangzhou Medical College. Written informed consent for participation was not required for this study in accordance with the national legislation and the institutional requirements.

## Author Contributions

CW analyzed data, wrote the first draft, and approved the final draft. JC and XH were responsible for collecting data, analyzing data, selecting research, and extracting data. MZ and YF were responsible for the analysis of pathological and immunohistochemical diagnoses. YW conducted study selection and statistical analysis. YL and WG proposed the scheme design, the concept, and the design of the study, and approved the final draft. All authors listed have made a substantial, direct, and intellectual contribution to the work and approved it for publication.

## Funding

This study was supported by the Medical Science and Technology Project of Zhejiang Province (Nos. 2020KY019, 2021PY036, 2016KYB011).

## Conflict of Interest

Author YW was employed by GE Healthcare.

The remaining authors declare that the research was conducted in the absence of any commercial or financial relationships that could be construed as a potential conflict of interest.

## Publisher’s Note

All claims expressed in this article are solely those of the authors and do not necessarily represent those of their affiliated organizations, or those of the publisher, the editors and the reviewers. Any product that may be evaluated in this article, or claim that may be made by its manufacturer, is not guaranteed or endorsed by the publisher.
