# Supplementary material for: Nomogram Based on CT Radiomics Features Combined With Clinical Factors to Predict Ki-67 Expression in Hepatocellular Carcinoma
Source: Front Oncol. 2022 Jul 6;12:943942. doi: 10.3389/fonc.2022.943942 (PMC9299359; doi:10.3389/fonc.2022.943942)
Supplement: Supplementary file 1 [file DataSheet_1.docx]

**Supplementary Material**

1. **Supplementary Methods**

**I. Acquisition of the contrast-enhanced CT images**

All patients were scanned using Siemens SOMATOM Definition AS 128 or Siemens SOMATOM Definition AS 40. Patients fasted for 6 hours and filled the gastrointestinal tract with 800 ~ 1000 milliliter water before scanning. 1.5ml/kg of the contrast agent Ultravist 370 was intravenously injected at a rate of 3.0-3.5 ml/s using a pump syringe (Medrad Vistron CT, American). CT images of arterial phase (AP) and portal vein phase (PVP) were collected after injection of contrast agent 35s and 70s, and then uploaded to Picture Archiving and Communication System (PACS).

**II. Radiomics Features Extraction**

**2.1 Derived images using wavelet and Laplacian of Gaussian (LOG) filters transformations**

Extract features from original and derived images using PyRadiomics. Derived images using two filters including wavelet filter and Laplacian of Gaussian (LOG) filters transformations to extract features. In addition to shape features extracted from original image. A LOG filter (σ=2.0, 3.0) was applied to decrease noise and enhance the edge detection. Most features were in compliance with feature definitions as described by the Imaging Biomarker Standardization Initiative (IBSI), which were available in a separate document by Zwanenburg et al. (1).

Wavelet: Wavelet filtering, yields 8 decompositions per level (all possible combinations of applying either a high or a low pass filter in each of the three dimensions. We used the discrete undecimated wavelet transform for decomposing the original images. The high-pass and low-pass wavelet functions were used in three axials; then, the original image could be decomposed into eight decompositions. We marked the original 3D images as 𝐺𝐺, the high-pass wavelet function as 𝐻𝐻 and the low-pass wavelet function as 𝐿𝐿. Them, the decompositions could be express as 𝐺𝐿𝐿𝐿, 𝐺𝐿𝐿𝐻, 𝐺𝐿𝐻𝐿, 𝐺𝐻𝐿𝐿, 𝐺𝐿𝐻𝐻, 𝐺𝐻𝐿𝐻, 𝐺𝐻𝐻𝐿, 𝐺𝐻𝐻𝐻. Specificity, the decomposition 𝐺𝐿𝐻𝐿 indicated that the original image was processed by using a low-pass filter, a high-pass filter and a low-pass filter in the x-axis, y-axis and z-axis, respectively. Based on these 8 decomposed images, histogram and textural features are extracted again. A number of 1037 features could be obtained through wavelet transform. The filters used for wavelet transform satisfy the perfect reconstruction conditions.

LoG: Laplacian of Gaussian filter, edge enhancement filter. Emphasizes areas of gray level change, where sigma defines how coarse the emphasised texture should be. A low sigma emphasis on fine textures (change over a short distance), where a high sigma value emphasises coarse textures (gray level change over a large distance). Applies a Laplacian of Gaussian filter to the input image and yields a derived image for each sigma value specified. A Laplacian of Gaussian image was obtained by convolving the image with the second derivative (Laplacian) of a Gaussian kernel.

The Gaussian kernel is used to smooth the image and is defined as

$$G(x,y,z,\sigma)=\frac{1}{\left( \sigma\sqrt{2\pi} \right)^{3}}ⅇ^{-\frac{x^{2}+y^{2}+z^{2}}{2\sigma^{2}}}$$

The Gaussian kernel is convolved by the laplacian kernel ∇^2 G(x,y,z), which is sensitive to areas with rapidly changing intensities, enhancing edges. The width of the filter in the Gaussian kernel is determined by σ and can be used to emphasize more fine (low σ values) or coarse (high σ values) textures.

All feature classes, in addition to the shape can be calculated on either the original image and/or derived image, obtained by applying one of several filters. The shape descriptors are independent of gray value, and are extracted from the label mask. If enabled, they are calculated separately of enabled input image types, and listed in the result as if calculated on the original image.

**2.2Standardization of data**

Extracted texture features were standardized, which removed the unit limits of the data of each feature and converted it into a dimensionless pure value. This allowed the indexes of different units or orders to be compared and weighted. We used a z-score normalization to make the image intensities fit a standard normal distribution with $\mu\text{=0}$ and $\sigma\text{=1}$, where $\mu$ is the mean value of the images, and $\sigma$ is the standard deviation. The normalized values (also called z-scores) of the image intensities (*x*) were calculated as follows:

$$z=\frac{x-\mu}{\sigma}$$

**III. Construction of the radiomics signature**

**3.1 Dimension reduction of radiomics features**

1037, 1037, and 2074 radiomics features were extracted from each patient of arterial phase (AP), portal vein phase (PVP), arterial phase combined portal vein phase (AVP), respectively. The features with ICC greater than 0.8 were selected, and the repeatability of the Spearman rank correlation test was used to exclude radiomics features with correlation coefficients lower than 0.70. Thus, there were 513, 519, and 1032 features retained respectively. Then, analysis of variance (ANOVA), Mann-Whitney U test, and correlation analysis were used to reduce data redundancy, preserving 21, 26, and 44 features respectively. Secondly, Gradient Boosting Decision Tree (GBDT) algorithm was used to reduce the dimension of the selected features, and 9, 11, and 16 features were retained.

**3.2 The GBDT algorithm**

GBDT is a combination of Gradient Boosting and Decision Tree. In case of GBDT algorithm, the weak learners are decision trees. Each tree attempts to minimize the errors of previous tree. By adding many trees in series and each focusing on the errors from previous one make boosting a highly efficient and accurate model. Since trees are added sequentially, boosting algorithms learn slowly. In statistical learning, models that learn slowly perform better. Gradient boosting algorithm combines the weak learners sequentially, so that each new learner adapts to the residuals of the previous step, thus improving the model. The final model aggregates the results from each step and a strong learner is achieved. To sum up, GBDT algorithm uses decision trees as week learners. A loss function is used to detect residuals.

GBDT is comprised of the following four steps:

1. Given the initial training data, the first base learner was trained.

2. Adjust the sample according to the performance of the base learner, and more attention would be put on the sample wrongly performed by the previous learner.

3. Use the adjusted sample, train the next base learner.

4. Repeat the above process T times, combining the weighted T learners. The training process of GBDT is shown in Figure S1, and its mathematical equation is as follows:

$$GBDT=\max_{s}\left[ \frac{1}{\left| S \right|}\sum_{f_{i}\in S} I(f_{i};c)-\frac{1}{\left| S \right|^{2}}\sum_{f_{i}f_{j}\in S} I(f_{i};f_{j}) \right]$$

In this study, after dimension reduction of GBDT, 9, 11 and 16 non-zero coefficient features are selected for AP, VP and AVP respectively. Details of the selected features are shown in Table S4.

**3.3 Definition of net benefit in the decision curve analysis (DCA)**

Decision curve analysis (DCA) was used to assess the clinical utility of the radiomics nomogram model in the two groups. The "true" positive and weighted false-positive rates were calculated across different threshold probabilities in the validation group to determine the net benefit. Specifically, the weighting factor was defined as the specific value of the threshold probability divided by 1 minus the threshold probability. A higher true-positive rate and a relatively low false-positive rate were suggested by a high net benefit. Plotting the net benefit against the threshold probability across the range of 0 to 0.8 generated the decision curve.

The net benefit was defined by the following equation:

Net Benefit = $TPR*\omega-\frac{P_{t}}{1-P_{t}}*FPR*\left( 1-\omega\right)$

𝑃𝑡 is the “threshold possibility” to stratify the patients into high-risk or low-risk groups. Patients with a probability of having high Ki-67 expression higher than 𝑃𝑡 are high-risk patients. These patients would be recommended for aggressive intervention, while low-risk would be referred to relatively mild treatment(s). TPR is the true positive rate, defined as the proportion of high-risk patients in the patients having high Ki-67 expression. FPR is the false positive rate, defined as the proportion of high-risk patients in the patients having low Ki-67 expression. 𝜔, is the prevalence of having high Ki-67 expression, calculated by dividing the total number of patients by the number of patients with high Ki-67 expression. In the condition of “treat none”, no patient is classified as high risk, both the TPR and FPR are zero, so the Net Benefit is zero. In the condition of “treat all”, all patients are classified as high risk (TPR=FPR=1), so the Net Benefit is calculated as

$${Net Benefit}_{Treat all}=\omega-\frac{P_{t}}{1-P_{t}}*\left( 1-\omega\right)=\frac{1-\omega}{P_{t}-1}+1$$

, which is a monotonically decreasing curve in the figure.

**IV. Radiomics signature calculation formula**

**Rad-score= Constant + coefficient *features**

Rad-score=0.0824-0.4028* log_sigma_2_0_mm_3D_firstorder_Skewness_AP

+0.0306* wavelet_LLH_firstorder_Mean_AP

-0.1495* wavelet_LLH_firstorder_Skewness_AP

+0.8197* wavelet_LHH_glcm_Correlation_AP

-0.6133* wavelet_HLH_glrlm_LowGrayLevelRunEmphasis_AP

+0.5036* wavelet_HLH_glszm_ZoneEntropy_AP

-0.6670* wavelet_HHL_firstorder_Kurtosis_AP

-0.3781* wavelet_HHH_glrlm_HighGrayLevelRunEmphasis_AP

+0.4237* wavelet_LLL_glszm_SmallAreaEmphasis_AP

-0.4658* log_sigma_2_0_mm_3D_glcm_Autocorrelation_PVP

-0.3164* wavelet_LLH_glcm_Autocorrelation_PVP

-0.2038* wavelet_LLH_glszm_SmallAreaEmphasis_PVP

-0.7730* wavelet_HLL_glcm_Imc1_PVP

+0.3954* wavelet_HHL_firstorder_Mean_PVP

-0.7368*wavelet_HHL_glszm_GrayLevel NonUniformityNormalized_PVP

+1.0907* wavelet_LLL_firstorder_Skewness_PVP

1. **Supplementary Figures and Tables**

**Supplementary Fig. S1** Patient collection route diagram

**Supplementary Fig. S2** Heatmaps of several models in the training and validation groups

**Supplementary** **Table S1** Detailed parameters of CT scanning

**Supplementary Table S2** Detailed radiomics features types

**Supplementary Table S3** Comparison of clinicopathological data and CT radiological features between the training and validation groups

**Supplementary Table S4** Remained radiomics features after dimension reduction

**Supplementary Table S5** Performance comparison of rad-score of three contrast-enhanced CT models in the training and validation groups

**Supplementary Table S6** Performance of different order radiomics features based on AVP CT images in the training and validation groups

**Reference:**

1.Zwanenburg A, Vallieres M, Abdalah MA, Aerts H, Andrearczyk V, Apte A, et al., The Image Biomarker Standardization Initiative: Standardized Quantitative Radiomics for High-Throughput Image-based Phenotyping. *Radiology* (2020) 295(2):328-38. doi: 10.1148/radiol.2020191145
