# Supplementary material for: Nomogram Based on CT Radiomics Features Combined With Clinical Factors to Predict Ki-67 Expression in Hepatocellular Carcinoma
Source: Front Oncol. 2022 Jul 6;12:943942. doi: 10.3389/fonc.2022.943942 (PMC9299359; doi:10.3389/fonc.2022.943942)
Supplement: Supplementary file 4 [file Table_1.docx]

**Table S1** Detailed parameters of CT scanning

| Parameters | Siemens SOMATOM Definition AS 128 | Siemens SOMATOM Definition AS 40 |
| --- | --- | --- |
| Tube voltage (kVp) | 120 | 120 |
| Tube current (mA) | Auto | Auto |
| Rotation times (s) | 0.5 | 0.5 |
| Field of view (mm) | 300-450 | 300-500 |
| Detector collimation (mm) | 128×0.625 | 40×0.625 |
| Pixel size | 512×512 | 512×512 |
| Slice thickness (mm) | 5 | 5 |
