# Supplementary material for: Nomogram Based on CT Radiomics Features Combined With Clinical Factors to Predict Ki-67 Expression in Hepatocellular Carcinoma
Source: Front Oncol. 2022 Jul 6;12:943942. doi: 10.3389/fonc.2022.943942 (PMC9299359; doi:10.3389/fonc.2022.943942)
Supplement: Supplementary file 5 [file Table_2.docx]

**Table S2** Detailed radiomics features types

| **Image types** | **Features types** | **Features number** |
| --- | --- | --- |
| Original | Shape-based (3D) | 14 |
|  | First Order Statistics | 18 |
|  | GLCM | 24 |
|  | GLDM | 14 |
|  | GLRLM | 16 |
|  | GLSZM | 16 |
|  | NGTDM | 5 |
| LoG filter | First Order Statistics | 18 |
| (2.0 sigma) | GLCM | 24 |
|  | GLDM | 14 |
|  | GLRLM | 16 |
|  | GLSZM | 16 |
|  | NGTDM | 5 |
| LoG filter | First Order Statistics | 18 |
| (3.0 sigma) | GLCM | 24 |
|  | GLDM | 14 |
|  | GLRLM | 16 |
|  | GLSZM | 16 |
|  | NGTDM | 5 |
| Wavelet filtering | First Order Statistics | 18 |
|  | GLCM | 24 |
|  | GLDM | 14 |
|  | GLRLM | 16 |
|  | GLSZM | 16 |
|  | NGTDM | 5 |
|  |  |  |

Note: LoG, Laplacian of Gaussian; GLCM, Gray Level Cooccurence Matrix; GLDM, Gray Level Dependence Matrix; GLRLM, Gray Level Run Length Matrix; GLSZM, Gray Level Size Zone Matrix; NGTDM, Neighbouring Gray Tone Difference Matrix.
