# Supplementary material for: Nomogram Based on CT Radiomics Features Combined With Clinical Factors to Predict Ki-67 Expression in Hepatocellular Carcinoma
Source: Front Oncol. 2022 Jul 6;12:943942. doi: 10.3389/fonc.2022.943942 (PMC9299359; doi:10.3389/fonc.2022.943942)
Supplement: Supplementary file 6 [file Table_3.docx]

**Table S3** Comparison of clinicopathological data and CT radiological features between training group and validation group

| **Variables** | **Training group (n = 120)** | **Validation group (n = 52)** | ***p* value** |
| --- | --- | --- | --- |
| Age (years) | 58.08±11.12 | 61.12±11.37 | 0.109 |
| Gender |  |  | 0.762 |
| Female | 12(10%) | 6 (11.5%) |  |
| Male | 108 (90%) | 46 (88.5%) |  |
| HBs-Ag |  |  | 0.16 |
| Negative | 25 (20.8%) | 16 (30.8%) |  |
| Positive | 95 (79.2%) | 36 (69.2%) |  |
| AFP(μg/L) |  |  | 0.487 |
| ≤20 | 53(44.2%) | 20 (38.5%) |  |
| ＞20 | 67 (55.8%) | 32 (61.5%) |  |
| Edmondson grade |  |  | 0.747 |
| I-II | 86(71.7%) | 36(69.2%) |  |
| III-IV | 34(28.3%) | 16(30.8%) |  |
| Tumor size |  |  | 0.059 |
| ≤5cm | 74 (61.7%) | 24 (46.2%) |  |
| ＞5cm | 46 (38.3%) | 28 (53.8%) |  |
| Cirrhosis |  |  | 0.167 |
| absent | 78 (65%) | 28 (53.8%) |  |
| present | 42(35%) | 24 (46.2%) |  |
| Tumor capsule | |  | 0.597 |
| Complete | 76(63.9%) | 31(59.6%) |  |
| Incomplete | 43(36.1%) | 21(40.4%) |  |
| Tumor margin | |  | 0.321 |
| Smooth | 67(56.3%) | 25(48.1%) |  |
| Non-smooth | 52(43.7%) | 27(51.9%) |  |

Note: HBsAg, serum hepatitis B surface antigen; AFP, alpha-fetoprotein.
