# Supplementary material for: Nomogram Based on CT Radiomics Features Combined With Clinical Factors to Predict Ki-67 Expression in Hepatocellular Carcinoma
Source: Front Oncol. 2022 Jul 6;12:943942. doi: 10.3389/fonc.2022.943942 (PMC9299359; doi:10.3389/fonc.2022.943942)
Supplement: Supplementary file 7 [file Table_4.docx]

| **Table S4.** Remained radiomics features after dimension reduction |
| --- |
| \| Sequences \| Features \| Category \| \| --- \| --- \| --- \| \| AP(n=9) \| log_sigma_2_0_mm_3D_firstorder_Skewness \| Firstorder \| \|  \| wavelet_HHH_glrlm_HighGrayLevelRunEmphasis \| GLRLM \| \|  \| wavelet_HHL_firstorder_Kurtosis \| Firstorder \| \|  \| wavelet_HLH_glrlm_LowGrayLevelRunEmphasis \| GLRLM \| \|  \| wavelet_HLH_glszm_ZoneEntropy \| GLSZM \| \|  \| wavelet_LHH_glcm_Correlation \| GLCM \| \|  \| wavelet_LLH_firstorder_Mean \| Firstorder \| \|  \| wavelet_LLH_firstorder_Skewness \| Firstorder \| \|  \| wavelet_LLL_glszm_SmallAreaEmphasis \| GLSZM \| \| PVP(n=7) \| log_sigma_2_0_mm_3D_glcm_Autocorrelation \| GLCM \| \|  \| wavelet_HHL_firstorder_Mean \| Firstorder \| \|  \| wavelet_HHL_glszm_GrayLevelNonUniformityNormalized \| GLSZM \| \|  \| wavelet_HLL_glcm_Imc1 \| GLCM \| \|  \| wavelet_LLH_glcm_Autocorrelation \| GLCM \| \|  \| wavelet_LLH_glszm_SmallAreaEmphasis \| GLSZM \| \|  \| wavelet_LLL_firstorder_Skewness \| Firstorder \|   Note. AP, arterial phase; PVP, portal vein phase; GLCM, Gray Level Cooccurence Matrix; GLSZM, Gray Level Size Zone Matrix; GLRLM, Gray Level Run Length Matrix. |
