# Supplementary material for: Nomogram Based on CT Radiomics Features Combined With Clinical Factors to Predict Ki-67 Expression in Hepatocellular Carcinoma
Source: Front Oncol. 2022 Jul 6;12:943942. doi: 10.3389/fonc.2022.943942 (PMC9299359; doi:10.3389/fonc.2022.943942)
Supplement: Supplementary file 8 [file Table_5.docx]

**Table S5** Performance comparison of radscore of three contrast-enhanced CT models

| **Models** | **Training group (n = 120)** | | |  | **Validation group (n = 52)** | | |  |
| --- | --- | --- | --- | --- | --- | --- | --- | --- |
|  | **AUC (95%CI)** | **Sensitivity** | **Specificity** |  | **AUC (95%CI)** | **Sensitivity** | **Specificity** |  |
| AP | 0.745(0.657-0.820) | 0.698 | 0.737 |  | 0.652(0.507-0.779) | 0.741 | 0.56 |  |
| PVP | 0.821(0.740-0.885) | 0.714 | 0.789 |  | 0.668(0.524-0.793) | 0.63 | 0.76 |  |
| AVP | 0.854(0.778-0.912) | 0.873 | 0.684 |  | 0.744(0.604-0.855) | 0.667 | 0.80 |  |
| Note: AP, arterial phase; PVP, portal venous phase; AVP, arterial phase combined with portal venous phase. | | | | | | | | |
