# Supplementary material for: Nomogram Based on CT Radiomics Features Combined With Clinical Factors to Predict Ki-67 Expression in Hepatocellular Carcinoma
Source: Front Oncol. 2022 Jul 6;12:943942. doi: 10.3389/fonc.2022.943942 (PMC9299359; doi:10.3389/fonc.2022.943942)
Supplement: Supplementary file 9 [file Table_6.docx]

**Table S6** Performance of different order radiomics features based on AVP CT images in the training and validation groups

| **Models** | **Training group (n = 120)** | | |  | **Validation group (n = 52)** | | |
| --- | --- | --- | --- | --- | --- | --- | --- |
|  | **AUC (95%CI)** | **Sensitivity** | **Specificity** |  | **AUC (95%CI)** | **Sensitivity** | **Specificity** |
| FSO | 0.730(0.651-0.802) | 0.571 | 0.807 |  | 0.631(0.497-0.757) | 0.481 | 0.76 |
| HO | 0.838 (0.777- 0.894) | 0.746 | 0.772 |  | 0.704 (0.578-0.821) | 0.704 | 0.68 |
| All | 0.854(0.778-0.912) | 0.873 | 0.684 |  | 0.744(0.604-0.855) | 0.667 | 0.80 |

Note: AVP, arterial phase combined with portal venous phase; FSO, first- and second-order; HO, high-order.
